# Identification of cervical squamous cell carcinoma feature genes and construction of a prognostic model based on immune-related features

**DOI:** 10.1186/s12905-022-01942-4

**Published:** 2022-09-03

**Authors:** Chun He, Lili Ren, Minchi Yuan, Mengna Liu, Kongxiao Liu, Xuexue Qian, Jun Lu

**Affiliations:** 1 General Practice Department, The First People’s Hospital of Jiashan, Jiaxing, Zhejiang People’s Republic of China; 2 Integrated TCM and Western Medicine Department, Cancer Hospital of The University of Chinese Academy of Sciences, Hangzhou, Zhejiang People’s Republic of China; 3 Medical Oncology Department, The First People’s Hospital of Jiashan, Jiaxing, Zhejiang People’s Republic of China; 4 Obstetrics and Gynecology Department, Lishui Hospital of Traditional Chinese Medicine, #800 Zhongshan Road 323000, Lishui, Zhejiang People’s Republic of China

**Keywords:** Cervical squamous cell carcinoma, Immune subtype, Prognostic model, Immune infiltration

## Abstract

As heterogeneity of cervical squamous cell carcinoma (CSCC), prognosis assessment for CSCC patients remain challenging. To develop novel prognostic strategies for CSCC patients, associated biomarkers are urgently needed. This study aimed to cluster CSCC samples from a molecular perspective. CSCC expression data sets were obtained from The Cancer Genome Atlas and based on the accessed expression profile, a co-expression network was constructed with weighted gene co-expression network analysis to form different gene modules. Tumor microenvironment was evaluated using ESTIMATE algorithm, observing that the brown module was highly associated with tumor immunity. CSCC samples were clustered into three subtypes by consensus clustering based on gene expression profiles in the module. Gene set variation analysis showed differences in immune-related pathways among the three subtypes. CIBERSORT and single-sample gene set enrichment analysis analyses showed the difference in immune cell infiltration among subtype groups. Also, Human leukocyte antigen protein expression varied considerably among subtypes. Subsequently, univariate, Lasso and multivariate Cox regression analyses were performed on the genes in the brown module and an 8-gene prognostic model was constructed. Kaplan–Meier analysis illuminated that the low-risk group manifested a favorable prognosis, and receiver operating characteristic curve showed that the model has good predictive performance. qRT-PCR was used to examine the expression status of the prognosis-associated genes. In conclusion, this study identified three types of CSCC from a molecular perspective and established an effective prognostic model for CSCC, which will provide guidance for clinical subtype identification of CSCC and treatment of patients.

**Supplementary Information:**

The online version contains supplementary material available at 10.1186/s12905-022-01942-4.

## Introduction

Cervical carcinoma is the third most common cancer in the developing country [1]. As a gynecological malignant tumor, cervical carcinoma ranks third in incidence among female malignancies with an increasing trend year by year, and its mortality ranks second [1, 2]. Cervical squamous cell carcinoma (CSCC) is the most common histological type of cervical carcinoma. The traditional therapeutic strategies including surgery, radiotherapy, chemotherapy, and targeted therapy have achieved great success, but some limitations remain yet. First, molecular-based clustering system for cervical carcinoma is remains imperfect, and specific targeted treatment is challenging [3]. Second, effective therapeutic strategies for patients with advance and recurrent cervical carcinoma are still insufficient. Therefore, further research on cervical carcinoma typing and finding biomarkers are necessary and vital for the accurate prediction and effective targeted treatment of cervical carcinoma.

Immunotherapy based on immune checkpoint inhibitors is becoming a hot topic in the field of cancer treatment [4]. Increasing evidence have suggested the correlation between immunotherapy and the abundance of immune cell infiltration [5, 6]. A study showed that higher number of infiltrating regulatory T cells in PD-L1 positive tumors pertains to better prognosis [7]. Moreover, the presence of PD-L1 + tumor-associated macrophages was associated with significantly poorer disease-free survival in cervical adenocarcinoma patients, which provides a reference for the application of PD-L1 in the immunotherapy of cervical carcinoma [8]. As the application of immunotherapy in cervical carcinoma treatment is related to immune cell infiltration in the TME, the identification of immune infiltration features has important reference value for evaluation of immunotherapy and development of prognostic features. ESTIMATE algorithm used in this study could comprehensively evaluate sample immune status according to gene profile, where Stromal Score, Immune Score, Estimate Score, and Tumor Purity would be scored for the tested sample.

In recent years, findings in molecular subtypes of cancer is providing strong support for the development of clinical treatment. Li et al. [9] identified and verified 6 molecular subtypes of squamous cell carcinoma with different molecular characteristics and clinical information. These subtypes represent different levels of immune cell infiltration, and the subtype dominated by M2 macrophage polarization is associated with immunosuppressive factors and indicates a poor prognosis. Zhang et al. [10, 11] explained the heterogeneity of breast cancer by the consensus clustering of methylation-related CpG islands in breast cancer dataset and identified 9 subtypes reflecting different DNA methylation patterns, including Luminal-B breast cancer related to local recurrence. In another study, triple-negative breast cancer is divided into 4 transcriptome-based subtypes, including luminal androgen receptor (LAR), immunomodulatory, basal-like immune-suppressed, and mesenchymal-like. This study revealed the drug treatments for each subtype and demonstrated the guidance of frequent aberrant mutations of CDKN2A oncogene and somatic cells in LAR subtype [12]. These results demonstrated the importance of revealing molecular subtypes for precise cancer treatment.

In the present study, weighted gene co-expression network analysis (WGCNA) was used to analyze the gene set in The Cancer Genome Atlas (TCGA) database. Together with ESTIMATE analysis, WGCNA provided gene modules with different expression patterns, as well as immune-related module genes. The genes in the module were classified into three immune subtypes by consensus clustering. Gene set variation analysis (GSVA), immune infiltration, HLA protein expression analysis, and ESTIMATE scores all supported the rationality of the subtyping, that is, each subtype represents a specific immune pattern. In addition, based on the regression and survival analyses of genes in the target module, this study constructed and validated the CSCC risk assessment model based and revealed the correlation between the model and immune cell infiltration. The classification of CSCC subtypes will lay a foundation for stratified clinical identification of CSCC and accurate prediction of disease recurrence, and the corresponding prognostic model can provide guidance for relating clinical immunotherapy.

## Materials and methods

### Data source

The mRNA expression transcript (in the form of FRKM) of CSCC in The Cancer Genome Atlas-Cervical Squamous Cell Carcinoma (TCGA-CESC) dataset were downloaded from TCGA database (https://portal.gdc.cancer.gov/) as well as clinical data, including 255 tumor samples and 2 normal samples.

### Analysis of immune microenvironment

Immune cells and stromal cells are two major non-tumor constituents in the tumor microenvironment (TEM). The evaluation of their expression is helpful to further evaluate the tumor purity in the tissue, showing a value for tumor diagnosis and prognostic assessment. The R-package ESTIMATE [13] was used to acquire the immune score and stromal score of tumor samples based on gene transcription. The indices related to cell immunity, including Immune Score, Stromal Score, ESTIMATE Score and Tumor Purity were obtained.

### Construction of WGCNA

WGCNA was constructed based on clustering genes with similar expression pattens and then merging them into modules; Subsequently, the modules of interest were screened via correlation analysis of module eigengene (ME) and indicators of interest. Analysis of target module genes and related traits was conducive to the screening of hub genes [14]. Firstly, 80% of genes in TCGA-CESC with 0 expression were eliminated, and the genes with the top 25% median absolute deviation (MAD) were retained. The sample clustering tree was constructed, and the outliers were removed. Finally, 4,553 genes from 253 samples were enrolled as the objects for subsequent research. The similarity matrix was constructed using Pearson correlation analysis and converted into the weighted adjacency matrix by power function. The range of soft threshold β was set to 1–20, and the scale-free topology criterion was that the correlation between average module connectivity (K) and p (k) was 0.85. The optimal soft threshold power was filtered to construct a scale-free topology network, and the adjacency matrix was converted into the topological overlap matrix (TOM). Finally, the lower limit of module gene was set to 50 and the threshold of dissimilarity was set to 0.25. Hierarchical clustering was carried out based on TOM dissimilarity to merge highly similar gene modules.

### Screening of gene modules highly related to TME and functional annotations

In principal component analysis of each module, the principal components were composed of MEs, which represent the gene expression profile of each module. To screen gene modules highly correlated with tumor immune-related features, four indicators, Immune Score, Stromal Score, ESTIMATE Score and Tumor Purity obtained by ESTIMATE algorithm were used as clinical traits to calculate the correlation coefficients between features and MEs of each module through Pearson correlation analysis. Finally, target modules significantly related to clinical features were screened. To further reveal the biological functions of genes in the target module, the genes were submitted to Metascape (http://metascape.org) database for functional analysis, and the default parameters were used [15–17].

### Consensus clustering analysis of samples

K-means consensus clustering analysis of TCGA-CESC samples was performed by using ConsensusClusterPlus package based on the expression matrix of the module genes [18]. To find the optimal clustering number K making the clustering results stable, a double sampling scheme was adopted with 80% sampling each time and 1,000 repetitions. The optimal clustering number was selected to further cluster TCGA-CESC tumor samples.

### GSVA and ssGSEA in different clustering groups

The differences in the biological pathways of different clustering sample groups were explored using the R-package GSVA for GSVA enrichment analysis [19]. Differential expression analysis (|logFC|> 0.1; FDR < 0.05) was performed using R-package limma. ssGSEA was performed on the mRNA data in different clustering groups using R-package GSVA. The immune cell gene set in this process was derived from the existing literature [20] and contains the mRNA expression of T cells, B cells, macrophages, natural killer cells, monocytes, etc.

### Construction of a prognostic model

Univariate Cox regression analysis was performed on the genes in the target module using R-package survival (*P* < 0.01). On this basis, the prognostic feature genes were obtained by Lasso and multivariate Cox regression analyses, and the multivariate regression model was constructed. Based on the expression level of each gene and the risk coefficient, the risk score of patients was calculated, and the prognostic risk assessment model was constructed.

1 $$Risk\;Score = \mathop \sum \limits_{i = 1}^{n} \exp_{i} *\beta_{i}$$

In the formula, n is the number of prognostic feature-related genes in model, exp_i_ is the expression of each feature gene, and β_i_ is the corresponding multivariate Cox regression coefficient of each feature gene.

Kaplan–Meier survival analysis was performed on high/low-risk groups with median risk score as the critical value [21]. The results were visualized using R-package survminer [22]. The receiver operating characteristic (ROC) curves were plotted using R-package survival ROC to calculate the AUC values of 1-, 3-, 5- year overall survival. Finally, the contents of B cells, CD8 + T cells, CD4 + T cells, dendritic Cells, neutrophils, and macrophages in each TCGA-CESC sample were obtained from the TIMER website (https://cistrome.shinyapps.io/timer/). Pearson correlation analysis was performed to analyze the correlation between risk scores and the degree of immune cell infiltration.

### Sample collection and qRT-PCR

The tumor and corresponding adjacent tissues of 15 cervical squamous cell carcinoma patients were collected from The First People's Hospital of Jiashan from May 2019 to May 2021. All the patients signed the informed consent. The collected tissues were stored at -80℃ to be processed by qRT-PCR assay.

Trizol (ThermoFisher, USA) was used to isolated total RNA. The extracted RNA was used to synthesize cDNA using PrimeScirpt RT reagent Kit (Takara, Japan). Based on the obtained cDNA, quantitative PCR was carried out by SYBR Green qPCR Master Mix (MedChemExpress, USA). GAPDH was selected as endogenous reference. The used primers were listed below (Table 1). The relative expression was calculated by 2^−ΔΔCT^ method.

**Table 1:** Primer list

GENE	PRIMERS
ISCU	F:ATATCGCCAAGGAGCTCTGC
	R:CTTCAGCCAGCACATCCAGA
MSMO1	F:GTTCCGAGGTTGGAACACCT
	R:TCTGGCTTATCCTGAACGGC
GCH1	F:TTGCGTACCTTCCTCAGGTG
	R:CCGGACAGACAGACAATGCT
EEFSEC	F:AACCAAGGCCAAGTTCCACA
	R:GATCTTCTTGGACTCGGGGC
SPP1	F:AGCAGCTTTACAACAAATACCCAG
	R:TACTTGGAAGGGTCTGTGGG
RHOG	F: GAGGGCACCAGGTCACTG
	R:CTCTGCGCGCTGTAATTGTC
LSP1	F:GGTTCAGGCTTCAGTCCCAG
	R:GGCCTGGGTGTATTGTTCCA
TCN2	F:GGTTCAGGCTTCAGTCCCAG
	R:GGCCTGGGTGTATTGTTCCA
GAPDH	F:AATGGGCAGCCGTTAGGAAA
	R:GCGCCCAATACGACCAAATC

## Results

### Construction of CSCC gene co-expression network and screening of immune-related module

**Fig. 1:**
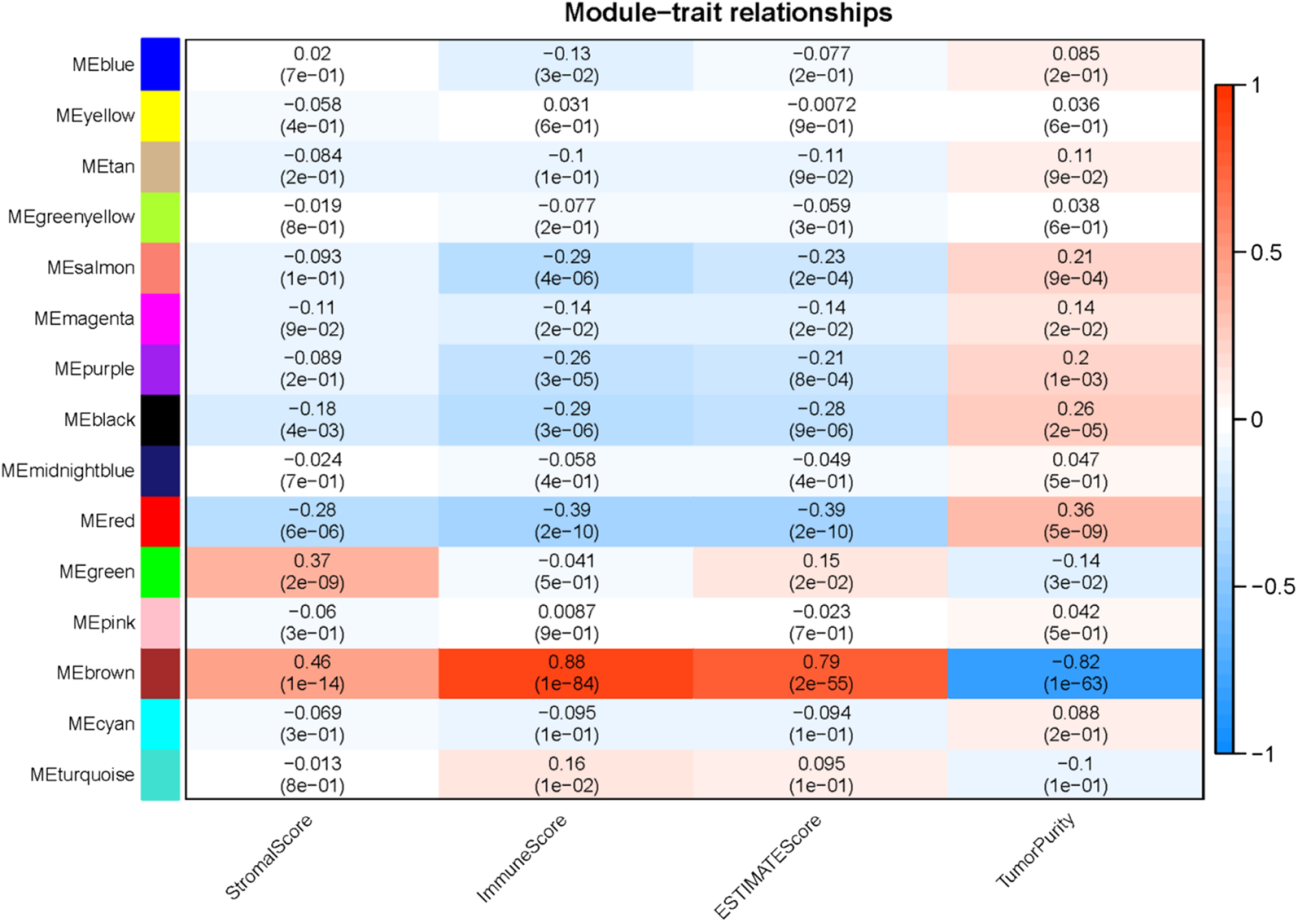
The immune-related modules based on WGCNA

Firstly, the unqualified samples and genes in the TCGA-CESC dataset were removed based on hierarchical clustering, and 253 samples and 4,553 genes were reserved for WGCNA to build the gene co-expression network. β = 3 (scale-free R2 = 0.92) was selected as an optimal soft threshold to construct a scale-free network, and finally 15 gene modules were obtained (Table 1). Then, the correlation between the feature genes of each module and four immune-related features (Stromal, Immune, Estimate Scores, and Tumor Purity) was calculated. It was found that brown module was significantly associated with immune status, presenting Immune Score (r = 0.88, *P* = 1e-84), Stromal Score (r = 0.46, *P* = 1e-14), ESTIMATE Score (r = 0.79, *P* = 2e-55) and Tumor Purity (r = -0.82, *P* = 1e-63) (Fig. 1). Therefore, the brown module was included in the subsequent study.

### Enrichment analysis of module genes

**Fig. 2:**
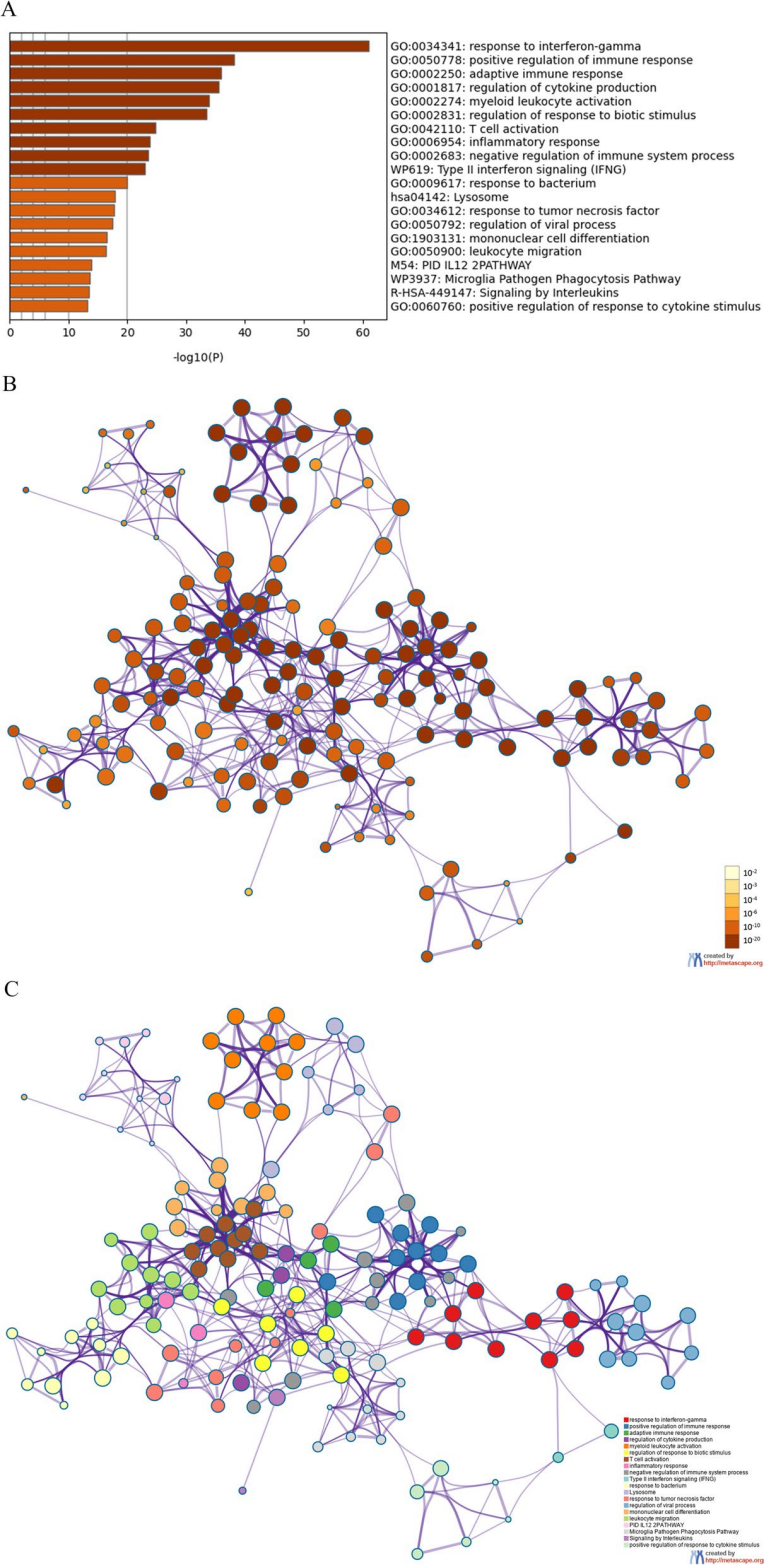
Enrichment analysis of genes in the brown module. **A** *P* value distribution of the top 20 enriched pathways and biological processes in the brown module; **B** The *P* value clustering network of genes in the brown module, with the redder the node color is, the more significant *P* value is; **C** Network analysis of enriched terms of genes in the brown module. Different node colors indicate different functional or pathway clusters that nodes belong to

Enrichment analysis was performed on 330 genes in the brown module to reveal relevant biological function. The results showed that the genes were largely enriched in the functions and pathways related to immune signal activation and immunomodulation, such as response to interferon-gamma, positive regulation of immune response, adaptive immune response, regulation of cytokine production, myeloid leukocyte activation, regulation of response to biotic stimulus, T cell activation, inflammatory response, negative regulation of immune system process, Type II interferon signaling (IFNG), Lysosome, response to tumor necrosis factor, regulation of viral process, etc. (Fig. 2A–C).

### Classification of tumor sample subtypes

**Fig. 3:**
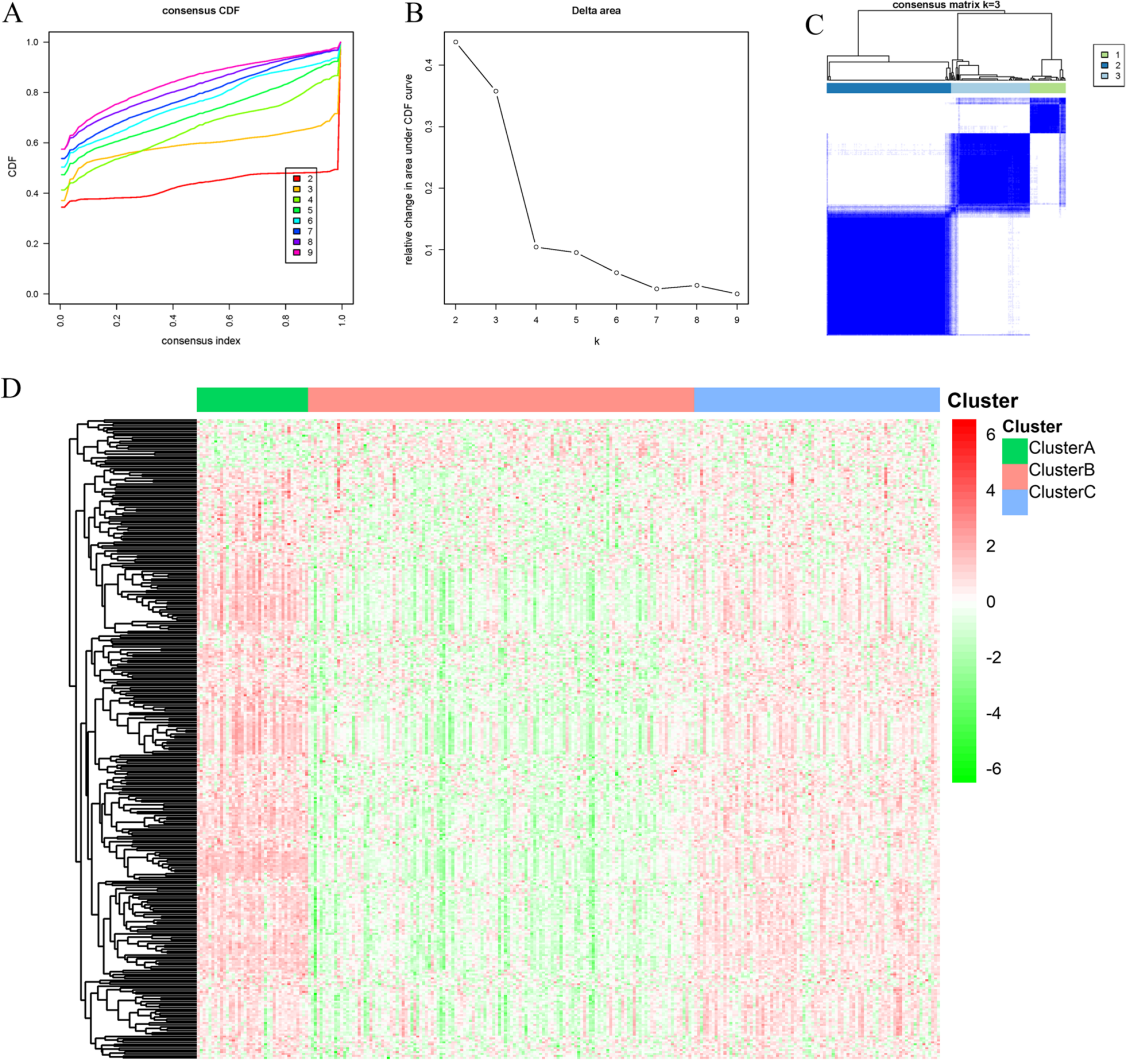
Consensus clustering analysis of gene expression pattern in the brown module. **A** Cumulative distribution function (CDF) of consensus clustering when K = 2 ~ 9; **B** Relative change of AUC of CDF curve when K = 2 ~ 9; **C** Tracking plot results of consensus clustering when K = 3; **D** Heat map of gene expression in different subtypes in the brown module

The brown module was known to be highly correlated with cellular immunity. In the present study, consensus clustering was conducted on tumor samples based on the expression patterns of genes in the brown module to identify different immune subtypes. Since the grouping was suboptimal when using K = 3 as the clustering value, we selected K = 3 to divide the samples into three groups (Fig. 3A–C). The samples obtained were named as cluster A (38 cases), cluster B (132 cases) and cluster C (84 cases). To better understand the immune patterns of the three subgroups, we explored the expression of genes in the brown module in the three subgroups (Fig. 3D). The results showed that most of the genes in the brown module were down-regulated in the cluster B subgroup, while most of the genes were up-regulated in the other two subgroups, and the overall level of gene up-regulation in the cluster A subgroup was more evident than that in the cluster C subgroup. Therefore, we assumed that the three subgroups might represent different immune patterns, which was further verified by subsequent analysis.

### GSVA of different tumor subtypes

**Fig. 4:**
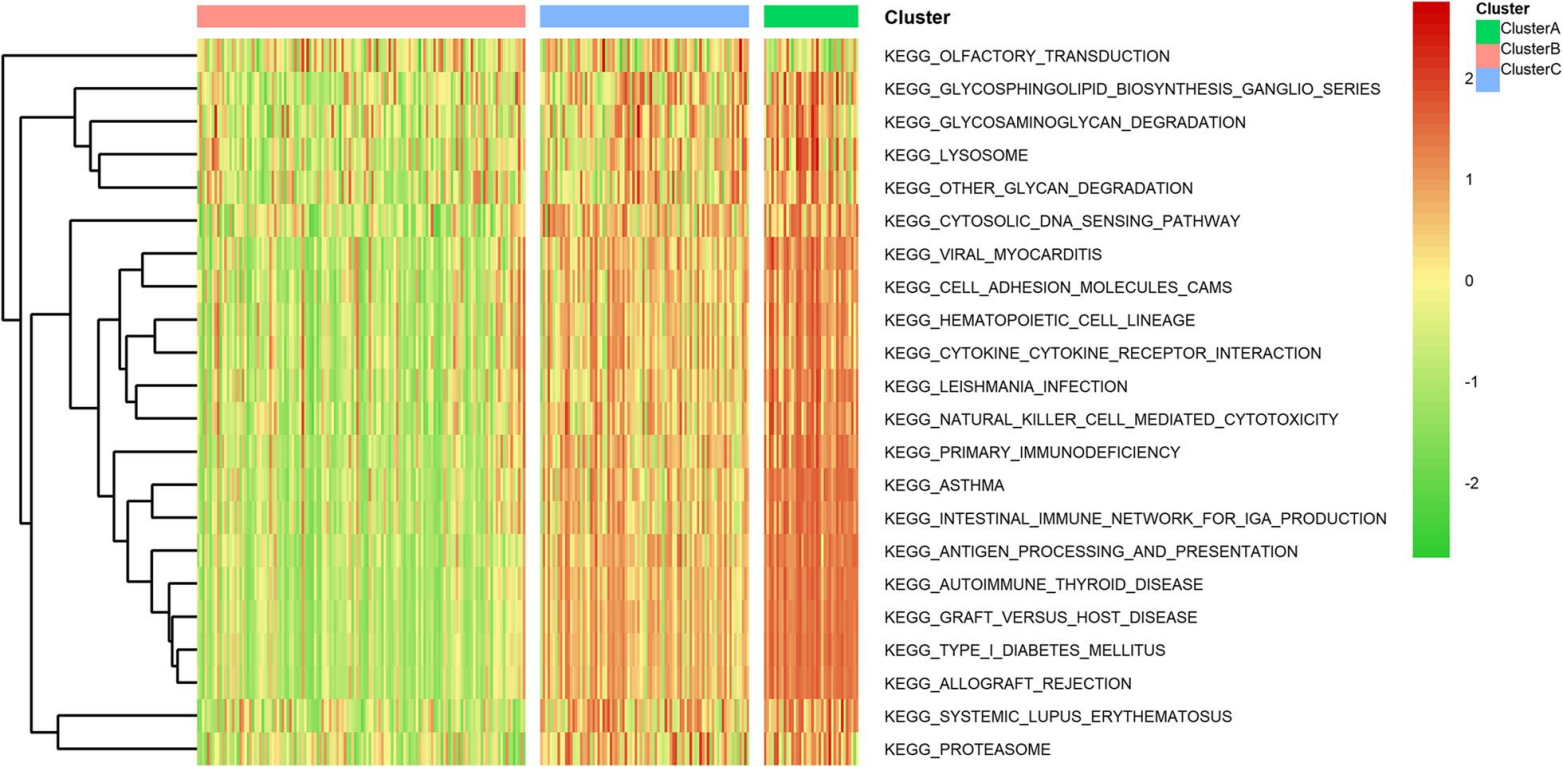
Heat maps of GSVA among different subtypes. Red: up-regulated pathways; green: down-regulated pathways

GSVA was done to explore the biological behaviors of the three tumor immune subtypes. Cluster A enriched in the pathways associated with immune deficiency and disease development, such as PRIMARY IMMUNODEFICIENCY, TYPE I DIABETES MELLITUS, INTESTINAL IMMUNE NETWORK FOR IGA PRODUCTION, ALLOGRAFT REJECTION, etc. Cluster B was enriched in pathways related to immunosuppressive biological processes. Cluster C was mainly enriched in pathways associated with cell adhesion, cytokine and cytotoxic activation pathways, including CYTOSOLIC DNA SENSING PATHWAY, CELL ADHESION MOLECULES CAMS, HEMATOPOIETIC CELL LINEAGE, CYTOKINE NATURAL KILLER CELL MEDIATED CYTOTOXICITY, CYTOKINE RECEPTOR INTERACTION, etc. (Fig. 4). These results indicated that the three subtypes have different enrichments in biological pathways, and it was speculated that these subtypes may have different biological behaviors.

### Immune feature analysis and classification validation of different tumor subtypes

**Fig. 5:**
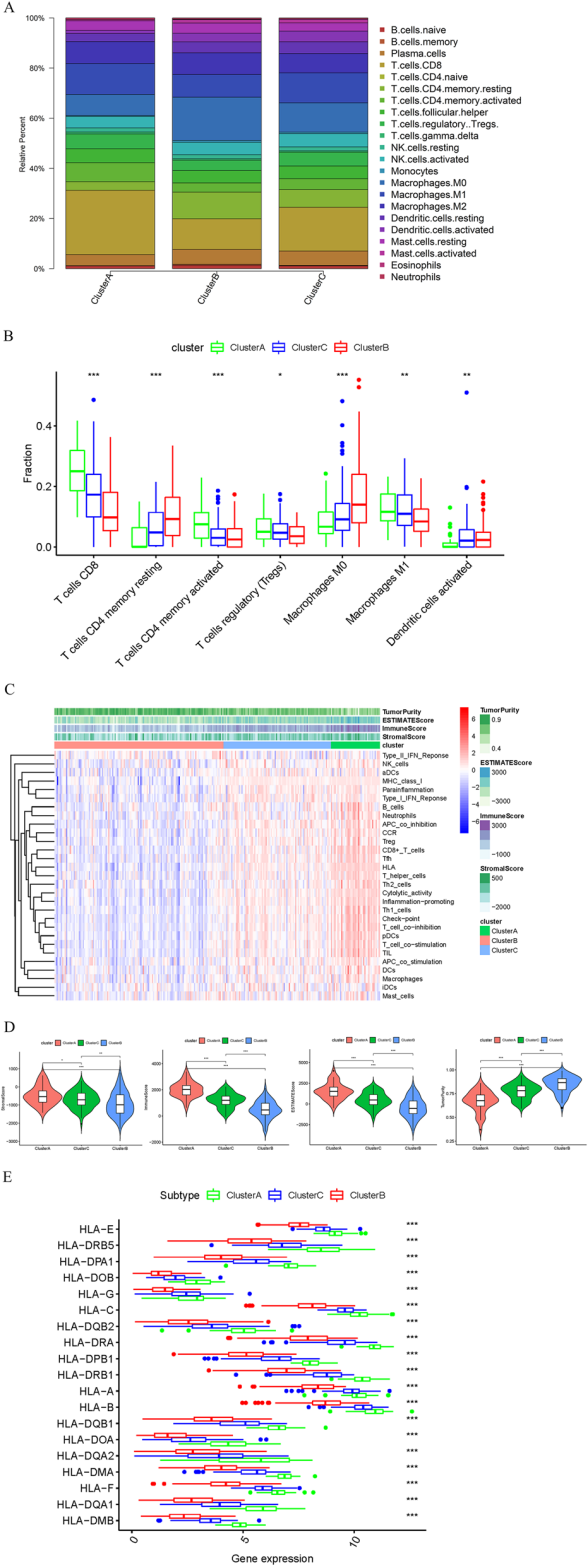
Analysis of immune cell infiltration and immune-related indices in different tumor subtypes. **A** CIBERSORT analysis of differences in immune cell composition among different subtypes; **B** Differences in the abundance of each immune infiltrating cell among different subtypes; **C** Heat map of immune cell typing; **D** Violin plot of the differential analysis of Tumor Purity, ESTIMATE Score, Immune Score and Stromal Score among the three subtypes; **E** Differences in the expression of HLA family gene among different subtypes

The analysis of cell infiltration in TME showed that there were differences in the contents of B cells, T cells, NK cells, monocytes and macrophages among the three subtypes (Fig. 5A). ssGSEA results showed significant differences in CD8 T cells, CD4 T cells, Treg cells, macrophage MD, M1 and dendritic cell contents among the three subtypes (Fig. 5B). To further verify the classification, ESTIMATE was used to calculate Stromal Score, ESTIMATE Score, Immune Score, and Tumor Purity based on mRNA data. These indicators were used to distinguish the high, low and medium immune groups. Compared with low immune cell infiltration group, the high immune cell infiltration group had lower Tumor Purity and higher Stromal Score, Immune Score and ESTIMATE Score. Therefore, Cluster A was defined as high immune group, Cluster B as low immune group, and Cluster C as medium immune group (Fig. 5C). High immune group was significantly positively correlated with ESTIMATE Score, Immune Score and Stromal Score, but negatively correlated with Tumor Purity (Fig. 5D). human leukocyte antigen (HLA) is an expression product of human major compatibility complex and is also a highly polymorphic allogeneic antigen [23]. In the present study, the correlation between immune cell infiltration and HLA family proteins in different subgroups was analyzed to verify the rationality of typing. The results demonstrated that the expression of HLA family gene was significantly downregulated in high immune group compared with in low immune group (Fig. 5E). The above results indicated that there were differences in the immune cell infiltration, immune-related scores and HLA family protein expression among subtypes, which also provided support for the rationality of the typing.

### Construction and assessment of prognostic model and analysis of immune infiltration

**Fig. 6:**
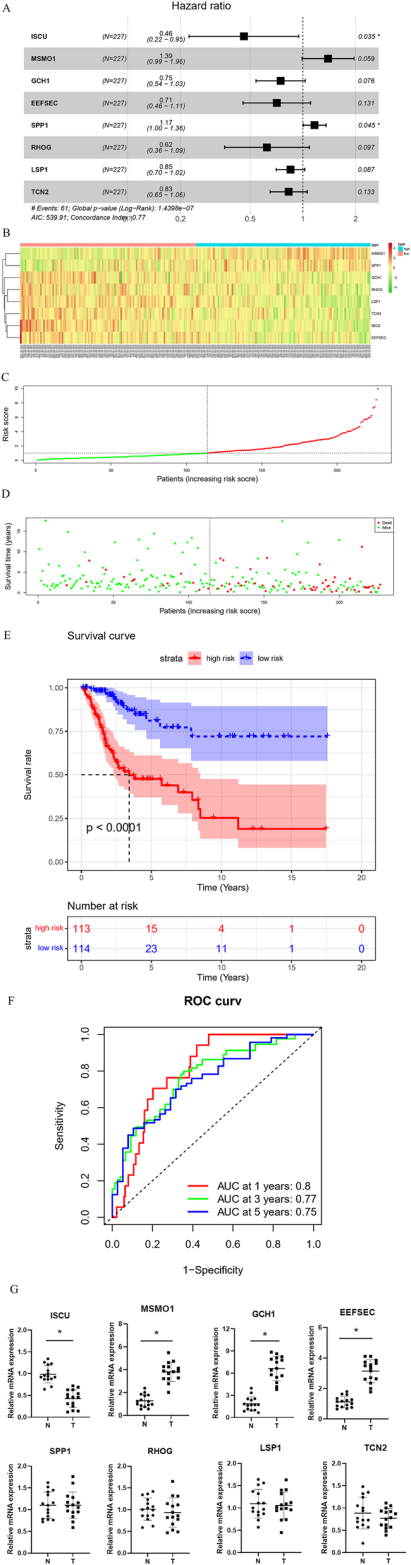
Construction and assessment of a prognostic model for CSCC. **A** Forest map of the 8-prognostic feature genes, *P < 0.05; **B** Heatmap of expression of the 8 prognostic feature genes in the high- and low-risk groups; **C** Risk score distribution of CSCC patients, with green representing the low-risk group and red representing the high-risk group; **D** Scatter plot of survival status of CSCC patients, with green and red dots representing survival and death, respectively; **E** Kaplan–Meier survival curve of the high- and low-risk groups; **F** ROC curves of the prognostic model predicting 1-, 3-, and 5-year overall survival of patients.; **G** qRT-PCR was used to measure the mRNA expressions of the feature genes

**Fig. 7:**
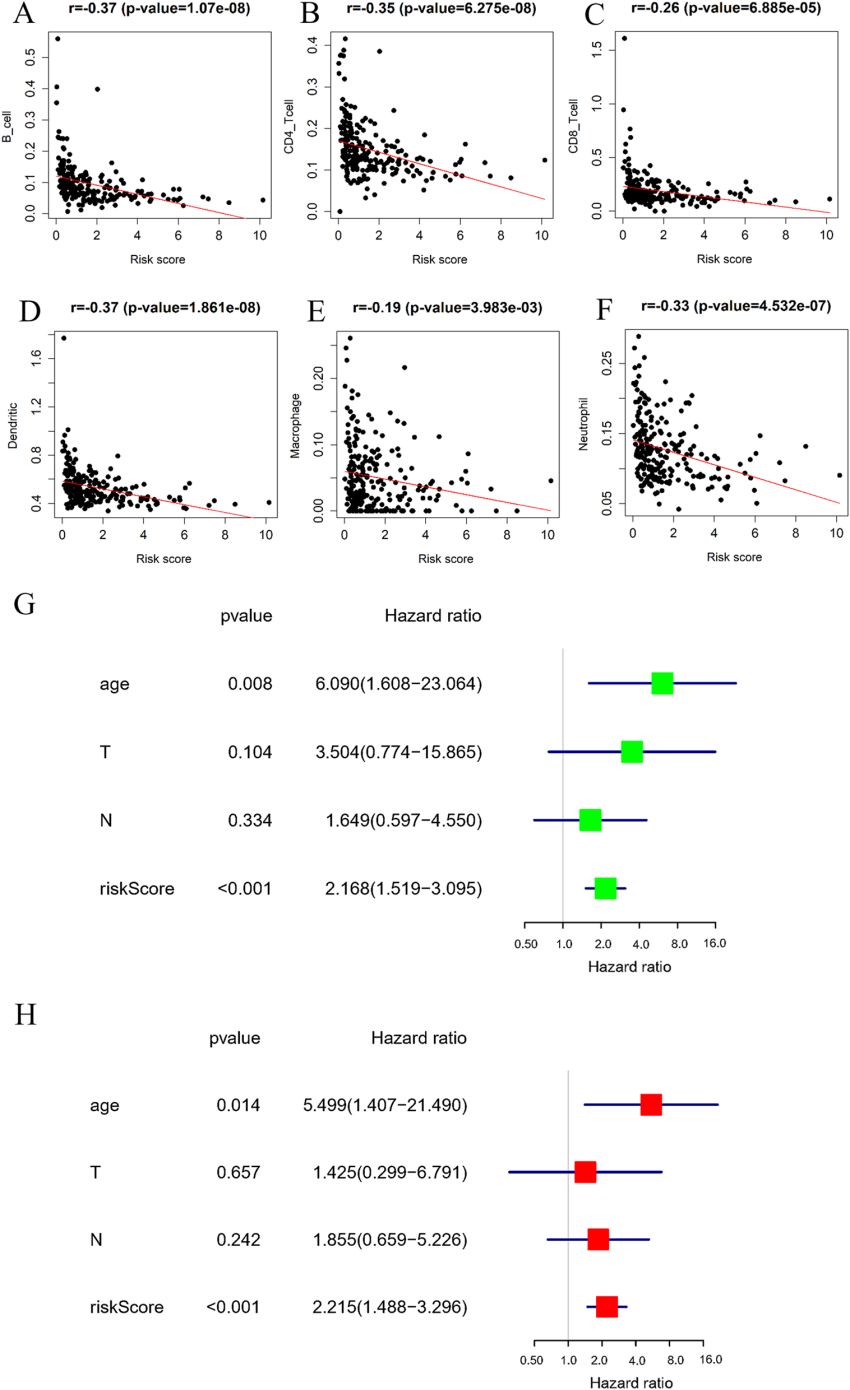
Correlation between risk score and infiltration degree of 6 immune cells. **A** Correlation between risk score and B_cell infiltration; **B** Correlation between risk score and CD4 T cell infiltration; **C** Correlation between risk score and CD8 T cell infiltration; **D** Correlation between risk score and dendritic cell infiltration; **E** Correlation between risk score and macrophage infiltration; **F** Correlation between risk score and neutrophil infiltration. **G**–**H** Univariate and multivariate Cox regression for risk score and the clinical features

Subsequently, a prognostic model was constructed based on the genes in the brown module. In the TCGA-CESC dataset, the samples with survival time less than 30 days were excluded. Then, for the 330 genes in the brown module, a univariate regression analysis was conducted, and 46 genes significantly associated with prognosis were obtained with *P* < 0.01 as the screening condition (Additional file 1: Table S1). Next, lasso and multivariate regression analyses were done on these 46 genes, and 8 feature genes were obtained finally, including ISCU, MSMO1, GCH1, EEFSEC, SPP1, RHOG, LSP1 and TCN2 (Fig. 6A, Additional file 2: Table S2). HRs of MSMO1 and SPP1 were higher than 1, which were risk factors for CSCC prognosis, while HRs of ISCU, GCH1, EEFSEC, RHOG, LSP1 and TCN2 were lower than 1, which could be protective factors for CSCC prognosis. The risk scores were calculated based on these 8 feature genes, and the samples were classified into high-risk and low-risk groups. According to the heat map, the expression levels of GCH1, EEFSEC, SPP1, RHOG, LSP1 and TCN2 were decreased overall with the increase of risk score (Fig. 6B). Based on the risk score distribution and survival time of the high/low-risk group samples, we found that the number of patients dying increased and the survival time decreased with the increase of risk score (Fig. 6C–D). Survival curves of the high/low-risk groups also demonstrated that patients in the low-risk group had a higher survival rate (Fig. 6E). ROC curve demonstrated the reliability of the risk assessment model in predicting 1-, 3- and 5- year survival rates of samples, with AUC values of 0.8, 0.77 and 0.75 respectively (Fig. 6F). Also, the expression statuses of the 8 genes were examined using qRT-PCR, whose results showed that ISCU was downregulated, while MSMO1, GCH1, EEFSEC were upregulated in the tumor tissues (Fig. 6G). In addition, this study assessed the correlation between the prognostic model and immune cell infiltration. As a result, the risk score was significantly negatively correlated with 6 immune cells, including B_ cell, CD8_ T cell, CD4_ T cell, neutrophil, dendritic cell and macrophage (Fig. 7A–F). To verify whether the risk score could be considered as an independent prognostic indicator, univariate and multivariate Cox regressions were introduced based on risk score and clinical features of the samples. As observed in Fig. 7G–H, risk score could independently serve as prognostic factor. In conclusion, we constructed an 8-feature gene risk assessment model to predict the prognosis of patients with CSCC and proved the favorable predictive ability of this model and revealed the association between the model and cellular immunity.

## Discussion

Immunotherapy is being used for a growing number of cancers. For example, Brahmer et al. [24] found that the efficacy and safety of nivolumab are better than docetaxel in treating patients with advanced squamous non-small cell carcinoma. Since high mutational burden in bladder cancer patients, immunotherapy, especially using anti-PD-1/PDL-1 antibodies, is understood as an optimal therapeutic option [25]. In KEYNOTE-045 phase 3 trial, anti-PD-1 immunotherapy evidently presented its strength on improving the prognosis of bladder cancer patients compared to chemotherapy [25]. It was also demonstrated that cancer immunotherapy needs to be improved in terms of pertinence. In view of the close correlation between TME and immunotherapy, it is necessary to systematically understand the tumor immune pattern [5, 6]. In the present study, a co-expression network of CSCC sample genes was constructed using WGCNA, and the brown gene module highly associated with immunity was screened by combining with immune traits was obtained from ESTIMATE. Subsequently, the samples were classified into three subtypes by consensus clustering based on their gene expression profiles. Due to the different expression patterns of genes in the module among subtypes, it was preliminarily revealed that the three subtypes may represent different immune patterns. The results provide reference for the recognition of CSCC immune pattern and may have important significance for the development of personalized immunotherapy.

The correlation between TME and the immune features of tumor subtypes had been demonstrated in a variety of cancers. Thorsson et al. [26] identified 6 immune subtypes: IFN-γ dominant, wound healing, inflammatory, immunologically quiet, lymphocyte depleted, and TGF-β dominant, which are established based on differences in macrophage and lymphocyte invasion levels, Th1:Th2, and tumor purity in the microenvironment. Another study evaluated the characteristics of molecular subtypes of colorectal cancer, including T cell infiltration and macrophage polarization, and identified the ability of subtypes to stratify disease [27]. To verify the differences in immune features between CSCC subtypes, this study assessed the infiltration of immune cells among subtypes, and divided the samples into high, medium, and low immune groups based on the immune traits calculated by ESTIMATE. The high immune group was significantly and positively correlated with ESTIMATE Score, Immune Score and Stromal Score but negatively correlated with Tumor Purity. In term of cancer therapy, HLA locus plays a key role in tumor recognition [28]. We also analyzed the expression of HLA family genes among subtypes. It was found that there were significant differences between groups, and the expression of the genes was significantly downregulated especially in the high immune group. Moreover, Li’s work performed similar result as we presented in our study [9], and based on his work, we additionally constructed a prognostic model. These results indicated the differences in immune features among the three CSCC subtypes and confirmed the rationality of these subtypes representing different immune patterns.

This study constructed a risk assessment model for CSCC based on 8 prognostic genes according to the MEs highly associated with immunity. MSMO1 and SPP1 can be used as adverse prognosis factors for CSCC, while ISCU, GCH1, EEFSEC, RHOG, LSP1 and TCN2 can be used as prognostic protective factors. MSMO1 is involved in the human cholesterol synthesis pathway and silencing of this gene is directly associated with decreased cell proliferation rate, especially in estrogen receptor-positive breast cancer [29]. SSP1 is associated with tumor immunity and can be directly related to the up-regulation of PD-1 to mediate macrophage polarization, thus promoting immune escape of tumor tissue [30]. ISCU is a gene associated with ferroptosis, and the downregulation of this gene in tissue is generally beneficial to tumor growth, which is consistent with the results of this study [31]. It was investigated that the inactivation of GCH1, a GTP cyclase hydrolase, seriously damages T cell activation in mouse and human immune systems [32]. EEFSEC may affect cancer risk by influencing human resistance to arsenic exposure [33]. Both RHOG and LSP1 are key regulators of cell proliferation and migration and play a critical role in regulating aggressive cancer cells, such as glioblastoma cells [34]. TCN2 plays a critical role in reversing the hypoxia microenvironment caused by cancer cells, which explains its existence as a protective factor for CSCC in this study [35]. In view of the functions of these genes in tumor immunity and cancer development, the present paper further investigated the correlation between the corresponding risk assessment model and cellular immunity. The results showed a negative correlation between the risk score and the infiltration of 6 types of immune cells, namely B cell, CD8 T cell, CD4 T cell, macrophage, dendritic cells and neutrophil. The high abundance of these cells in the immune microenvironment is generally associated with favorable prognosis and immunity [36–38]. Taking T cells as an example, it is generally regarded as a typically key participant in immune checkpoint blocking therapy [39]. Cancer immunotherapies such as vaccines and checkpoint blocking can promote the autoimmune response of the body and enhance T and B cell responses [40, 41]. Therefore, the regulation of the 8 immune-related genes identified in the present study may lead to poor prognosis of patients by linking immunosuppression.

However, this study was a pure bioinformatics analysis and had limitations. Firstly, data acquisition was completely dependent on public databases. This inevitably led to systematic errors between databases, so more accurate RNA sequencing is needed for verification. Secondly, this study found three CSCC subtypes that could represent different immune patterns but lacked experiments in vitro to verify the immune behavior of each type. Finally, even if the CSCC risk assessment model was found to be related to the infiltration of immune cells, this study cannot prove that the prognostic genes can cause poor prognosis through immunosuppression, which requires a combination of experimental basis and clinical evidence.

In conclusion, this study screened the immune-related gene set associated with CSCC using a gene co-expression network combining ESTIMATE analysis. Based on consensus clustering of gene sets, the samples were divided into three subtypes representing independent immune patterns. A CSCC prognostic risk assessment model was constructed and validated on the basis of regression analysis, survival analysis and immune infiltration analysis of related gene sets. However, several limitations remain in our study, for one of the concerned points is lack of clinical validation. To validate in clinical manner, we are planning to establish bladder cancer sample library for further research. These results will provide guidance for accurate classification and clinical treatment of CSCC.

## Supplementary Information

**Additional file 1**. **Table S1**: Univariate Cox regression analysis of prognosis-related genes from targeted modules of WGCNA

**Additional file 2**. **Table S2**: Lasso and Multivariate Cox regression analyses of prognosis-related genes from targeted modules of WGCNA

## Data Availability

The data used to support the findings of this study are included within the article.
